# Identification of differentially expressed genes associated with ferroptosis in ulcerative colitis

**DOI:** 10.1371/journal.pone.0327990

**Published:** 2025-07-29

**Authors:** Fang Zhang, Xin Jiang, Xuyu Chen, Zheng Wang, Jianlei Xia, Bingcheng Wang, Mei Wang, Yanbing Ding

**Affiliations:** 1 Department of Gastroenterology, The Affiliated Hospital of Yangzhou University, Yangzhou University, Yangzhou, China; 2 Department of outpatient, Jinling Hospital, The Affiliated Hospital of Nanjing University, Nanjing, China; 3 Department of Pathology, The Affiliated Hospital of Yangzhou University, Yangzhou University, Yangzhou, China; The Second Affiliated Hospital of Guangzhou Medical University, CHINA

## Abstract

**Purpose:**

To identify ferroptosis-related genes associated with the development of Ulcerative colitis (UC), through bioinformatics and basic experiments.

**Methods:**

Ferroptosis-related genes were identified from UC microarray data extracted from the GEO database and FerrDb. GO and KEGG pathway enrichment analyses were performed. Hub genes were identified through PPI analysis, leading to TF-hub gene and miRNA-hub gene regulatory network, predicting potential drug candidates by DSigDB. RT-qPCR, WB and IHC were employed to validate hub gene expression in animal samples. Clinical samples were gathered from Normal examiners and UC patients and IHC was performed to verify SLC7A5.

**Results:**

Eleven ferroptosis-related DEGs were identified (nine upregulated and two downregulated genes) in UC, with eight genes chosen from the PPI network. MCC algorithm demonstrated that SLC7A11, PSAT1, SLC7A5, ACSF2, and ACSL4 were hub genes, predicting TFs, miRNAs and drugs. RT-qPCR confirmed significant differential expression of SLC7A5, ACSL4, and ACSF2. WB and IHC of mouse samples, as well as IHC of clinical samples, revealed significantly elevated SLC7A5 expression in the UC group compared to controls.

**Conclusion:**

SLC7A5 emerged as a potential focus for understanding UC pathogenesis, potentially influencing ferroptosis. FOXP3, STAT6, and hsa-miR-186-5p are implicated in UC and ferroptosis, with minocycline identified as a potential treatment by inhibiting ferroptosis.

## 1 Introduction

Inflammatory bowel disease (IBD), encompassing Crohn’s disease (CD) and ulcerative colitis (UC), is a chronic inflammatory disorder with an uncertain etiology [1]. In recent decades, the epidemiology of inflammatory bowel disease (IBD) has significantly changed, with rising incidence rates observed across all age groups in developed countries and a notable increase in Asia and other emerging and developing nations, indicating a global spread of the disease [2]. As a subtype of inflammatory bowel disease, ulcerative colitis (UC) is characterized by chronic inflammation of the colonic mucosa, primarily affecting the rectum and sigmoid colon [3]. The worldwide prevalence of UC ranges from 5.5 to 24.3 cases per 100,000 people [4]. Its pathogenesis is driven by a complex interplay of genetic factors, immune system abnormalities, environmental influences, mucosal barrier dysfunction, psychological stress, and gut dysbiosis [5,6]. However, the precise pathogenesis of UC remains incompletely elucidated. Thus, investigating the underlying etiology and mechanisms contributing to UC is crucial for enhancing diagnostic and therapeutic approaches for this condition.

Recently, ferroptosis has emerged as a notable iron-dependent cell death mechanism distinct from apoptosis. Ferroptosis primarily manifests through two pathways—exogenous and endogenous—characterized by a dysregulated cellular REDOX system [7]. Dysfunctions such as reduced cysteine uptake and inhibition of glutathione peroxidase-4 (GPX4) can trigger ferroptosis [8]. Under aberrant iron metabolism, the normal regulation of free radical and lipid peroxide production and consumption by various redox-active enzymes is disrupted, leading to the accumulation of radicals and peroxides, cellular structural damage, and eventual cell demise, thereby contributing to disease progression [9,10]. Recent evidence indicates that the modulation of ferroptosis plays a significant role in influencing the clinical progression of ulcerative colitis (UC), underscoring its close relationship with the pathology of the disease [11]. Hence, elucidating the ferroptosis pathway’s mechanistic role in UC pathogenesis holds significant therapeutic promise.

In this study, we performed a comprehensive integration of differentially expressed genes (DEGs) associated with UC sourced from the GEO database, along with ferroptosis-related genes obtained from FerrDb, to tackle the shortage of novel pharmacodynamic markers or biomarkers specific to ferroptosis [12]. This approach enabled us to successfully identify DEGs implicated in ferroptosis. We subsequently validated these DEGs using an independent dataset from GEO and further investigated potential biomarkers through experiments conducted with a DSS-induced mouse model and clinical samples from UC patients. The primary objective of this research was to identify potential biomarkers that could serve as viable pharmacological therapeutic targets for UC, thereby advancing the understanding and treatment of this condition.

## 2 Materials and methods

### Source of data acquisition

**Fig 1:**
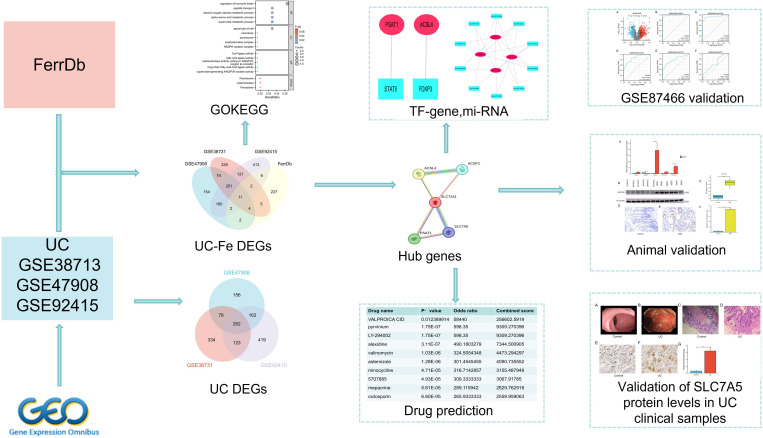
Flowchart illustrating the study workflow, outlining data collection, filtering, processing, and analysis. The study emphasizes the investigation of DEGs, particularly those related to ferroptosis, along with the exploration of Gene Ontology (GO), KEGG pathways, transcription factors (TF), miRNA, and drug prediction. Furthermore, the validation phase encompasses the assessment of datasets, animal models, and clinical samples.

UC-related microarray sequencing datasets were retrieved from the Gene Expression Omnibus (GEO) database (https://www.ncbi.nlm.nih.gov/geo/) for analysis in the present study, using the keyword ‘ulcerative colitis’ [13]. Four GEO datasets related to ulcerative colitis (UC) were meticulously selected for inclusion in our study: GSE38713, GSE47908, GSE92415, and GSE87466. The following screening criteria were applied: (i) samples from human tissues; (ii) the dataset contained microarray expression data; (iii) the dataset included samples from both UC patients and healthy controls; (iv) the total number of samples was > 10; and v) the number of differentially expressed genes (DEGs) was > 100. Consequently, four GEO datasets, GSE38713, GSE47908, GSE92415, and GSE87466 (Table 1), were included in this study. GSE38713, sourced from Spain, consists of RNA extraction and Affymetrix microarray hybridization results from intestinal mucosa samples, including 13 healthy controls, 8 patients with inactive UC, 7 with unaffected active UC, and 15 with affected active UC, utilizing the Affymetrix Human Genome U133 Plus 2.0 Array (GPL570). GSE47908, collected from Denmark, features colonic RNA sequencing data from 60 subjects, consisting of 45 UC patients (20 with left-sided colitis, 19 with pancolitis, and 6 with UC-associated dysplasia) and 15 healthy controls, also employing the Affymetrix Human Genome U133 Plus 2.0 Array (GPL570). GSE92415, obtained from the USA, provides gene expression profiles for 183 individuals, including 162 UC patients and 21 normal controls, analyzed using the Affymetrix HT HG-U133 + PM Array Plate (GPL13158). Finally, GSE87466, also from the USA, includes expression data of colonic mucosa from 47 individuals, comprising 22 healthy controls and 25 UC patients, utilizing the same Affymetrix HT HG-U133 + PM Array Plate (GPL13158). Ferroptosis-related genes were identified from the FerrDb database (http://www.zhounan.org/ferrdb/index.html), and a total of 259 genes were found. The overall research process of the present study is illustrated in Fig 1.

**Table 1:** Characteristics of the included datasets.

Dataset ID	Country	Platforms	No. of samples	Affymetrix Gene Chip
GSE38713	spain	GPL570	13 Healthy controls 30 UC	Affymetrix Human Genome U133 Plus 2.0 Array
GSE47908	Denmark	GPL570	15 Healthy controls 45 UC	Affymetrix Human Genome U133 Plus 2.0 Array
GSE 92415	USA	GPL13158	21 Healthy controls 162 UC	Affymetrix HT HG-U133 + PM Array Plate
GSE87466	USA	GPL13158	21 Healthy controls 87 UC	Affymetrix HT HG-U133 + PM Array Plate

### Identification of ferroptosis‑related DEGs in UC

The R software version 4.2.1(released on June 9, 2021) was utilized in combination with DESeq2 (version 1.34.0) and limma (version 3.52.2), to conduct a comprehensive analysis of differential gene expression (DGE) across GSE38713, GSE47908, and GSE92415 datasets. For the screening of differentially expressed genes (DEGs), we applied stringent criteria of |log2FoldChange| ≥ 1.0 and an adjusted P-value < 0.05, ensuring robust identification of significant gene expressions. Similarly, using R version 4.2.1, the differential analysis results were visualized through volcano plots from ggplot2 (version 3.4.4) and heatmaps from ComplexHeatmap (version 2.13.1), while the overlap of ferroptosis-related genes across the datasets was assessed using the VennDiagram package.

### GO and KEGG pathway analysis

Enrichment analyses were conducted using the “clusterProfiler” package in R software, focusing on Gene Ontology (GO) and Kyoto Encyclopedia of Genes and Genomes (KEGG) pathways. Criteria for significance included a strict threshold of p < 0.25 and adjusted p values (padjust) < 0.05. Furthermore, Pearson correlation tests were employed to explore mRNA level correlations, providing insights into the interactions among genes implicated in UC.

### Constructing a protein‑protein interaction (PPI) network and hub gene selection

Protein-Protein Interaction (PPI) networks are crucial for understanding biological mechanisms and protein interactions, providing valuable insights into molecular functions. In our study, we utilized STRING software (version 11.0) to analyze differentially expressed genes, setting a median confidence score threshold of 0.7 for network reliability. The resulting PPI network was visualized using Cytoscape software (version 3.7.2) to enhance understanding of its architecture, with node size and color determined by topological analysis scores, illustrating each gene’s significance and connectivity.

Employing CytoHubba, a robust Cytoscape plug-in renowned for its unique network metrics, our study focused on identifying pivotal regulators within biological networks. This facilitated a nuanced exploration of key drivers orchestrating complex biological processes. CytoHubba played a critical role in finely screening PPI network modules to pinpoint pivotal nodes and discern key genes. Leveraging the MCC algorithm, five genes were selected for comprehensive analysis based on their significance. A gradient color scheme from red to yellow was adopted to visually represent the importance of these core genes, offering a clear and intuitive depiction.

### Potential transcription factors and miRNAs prediction

Enrichr (http://amp.pharm.mssm.edu/Enrichr), an open network services platform, played a pivotal role in our research by facilitating multi-gene set enrichment analysis. We identified common differentially expressed genes from our dataset and investigated associated transcription factors, which play a crucial role in regulating gene expression by modulating chromatin structure and transcription processes through DNA sequence recognition and binding. Additionally, we utilized the miRTarBase module within Enrichr to explore gene-miRNA interactions. miRTarBase, a comprehensive miRNA-target interaction database, provided valuable insights into miRNA-mediated regulation of protein expression by influencing mRNA stability and translation efficiency, which are essential in biological processes. To visualize these interactions, we employed Cytoscape software to construct interaction networks among genes, transcription factors (TFs), and miRNAs.

### Potential target drug prediction

To anticipate potential drugs targeting the five hub genes associated with ulcerative colitis (UC), predictive analysis was conducted using the DSigDB module within the Enrichr platform. Specifically, DSigDBv1.0 (http://dsigdb.tanlab.org/DSigDBv1.0/) was utilized to forecast potential drugs targeting these hub genes. Subsequently, the top 10 scores were identified and designated as candidate drugs.

### Animals and establishment of DSS-induced colitis model

Twelve female wild-type C57BL/6 mice, aged 8–10 weeks and certified as specific pathogen-free (SPF), were obtained from the Yangzhou University Center for Comparative Medicine in Yangzhou, China. Prior to the experiment, the mice underwent a minimum one-week acclimatization period with ad libitum access to food and water. They were housed in groups of three per cage in an enriched environment, maintained at a room temperature of 22°C on a 12-hour light/dark cycle, and provided with standard chow (1010001, Jiangsu Xietong Pharmaceutical Bio-engineering Co., Ltd, China).

When conducting research involving mice, it is essential to adhere to the NC3Rs guidelines concerning humane endpoints. To ensure animal welfare, humane endpoints were established. Criteria for Humane Endpoints included severe weight loss exceeding 15% of baseline body weight, significant distress or pain unresponsive to analgesics, inability to consume food or water, pronounced lethargy, severe weakness, dehydration, visible distress, and abnormal behaviors such as labored breathing. Once a mouse meets the humane endpoint criteria, it is essential to act promptly. The time before euthanasia should be minimized to reduce suffering. Throughout the study, all animals were closely monitored, and no mice died prior to reaching the established euthanasia criteria.

The C57BL/6 mice were randomly divided into two groups: a control group (n = 6) receiving standard drinking water, and a DSS group (n = 6) receiving drinking water containing 3.0% (w/v) dextran sulfate sodium (DSS, molecular weight 36–50 kDa, catalog number 60316ES60, YEASEN) for 7 days to induce colitis. Trained personnel monitored the health and behavior of all mice daily, assessing parameters such as body weight, food and water intake, activity levels, stool consistency, and the presence of blood in the stool. Animal welfare considerations were thoroughly addressed, including efforts to minimize suffering and distress through the use of analgesics and anesthetics as appropriate, along with special housing conditions to ensure a comfortable environment for the animals throughout the study. All staff involved in animal care have received training on recognizing signs of distress and humane euthanasia procedure. A disease activity index (DAI) (Table 2) was calculated daily using a 0–4 scoring system based on predefined parameters.

**Table 2:** Method for Calculating DAI.

Score	Score 0	Score 1	Score 2	Score 3	Score 4
Weight Loss	No apparent decrease	Slight decrease	Moderate decrease	Marked decrease	Severe decrease
Stool	<5%	(1%–5%)	(5%–10%)	(10%–20%)	(>20%)
Consistency	Normal	Slightly soft	Mucoid	Watery	Bloody
Rectal Bleeding	None	Slight (light blue)	Mild (blue)	Severe(dark blue)	Gross visible blood

DAI = Score for Weight Loss + Score for Stool Consistency + Score for Rectal Bleeding.

At the conclusion of the experimental procedures, humane euthanasia was performed on the mice via intraperitoneal injection of pentobarbital-phenytoin solution as per American Veterinary Medical Association (AVMA) guidelines. Mice were monitored for 10–15 minutes post-injection to confirm euthanasia (all mice were confirmed deceased), assessed by the cessation of respiration, absence of cardiac activity, and fixed, dilated pupils. Biological materials and carcasses were disposed of in accordance with institutional hazardous waste management protocols, ensuring compliance with biohazard safety standards and the maintenance of animal welfare.

### Ethical statement

All animal experimental protocols were performed in accordance with relevant guidelines and regulations, including ARRIVE guidelines and the Animal Ethics Committee of Yangzhou Medical University. The animal study was reviewed and approved by Ethics Committee of Experimental Animal Center, Yangzhou University School of Medicine (approval no. YXYLL-2024–077). In this study, we implemented measures to minimize animal usage while ensuring optimal welfare conditions throughout all phases of the experiments. Mice were monitored daily to assess their general well-being, and their body weight was recorded on a weekly basis. All behavioral assessments were conducted during the light cycle, with the observer blinded to the experimental treatments assigned to the animals.

Human related experiments were reviewed and approved by the Ethics Committee of the Affiliated Hospital of Yangzhou University (approval no. 2022-YKL7). Written informed consent was obtained from the patients for inclusion of their samples in the biobank.

### UC and control samples from patients

The Ethics Committee of Yangzhou University Affiliated Hospital approved the research project titled “Establishment and Application of a Biological Sample Database for Diagnosis of Intestinal Diseases” (Approval No. 2022-YKL7), following a comprehensive review. The project is scheduled from July 2022 to July 2027. Data access for research purposes was conducted on May 31, 2024. For the study, a total of 12 tissue samples were meticulously selected from the patient biobank. The sampling technique employed was a stratified random sampling method to ensure a balanced representation of the target population. Specifically, we included 6 samples from patients diagnosed with ulcerative colitis (UC) and 6 samples of normal intestinal tissue. These samples were collected within the timeframe of July 2022 to May 2024, ensuring that all tissues were obtained under standardized protocols to minimize variability. All participants provided written informed consent prior to inclusion, and the authors had access to identifiable participant information during and after the data collection period. Immunohistochemical experiments were performed in June 2024.

### Hematoxylin and eosin (H&E) staining

The tissue specimens were initially fixed in 10% buffered formalin for 48 hours at 4°C. Subsequently, they were embedded in paraffin and sectioned at a thickness of 4 µm. The slides then underwent dewaxing twice in 100% xylene for 30 minutes at 56°C and were rehydrated through a series of graded alcohol solutions (100%, 90%, 80%, 70%, and 50%). After a 5-minute rinse in H2O, the slides were stained with H&E (cat. no. G1121; Solarbio) for 10 minutes at room temperature. Post-staining, the sections were dehydrated in ethanol, cleared in 100% xylene, and mounted with Permount (Wuhan Servicebio Technology Co., Ltd.) at room temperature.. Visualization was performed under a TE2000 microscope (Nikon Corporation of Japan) with white light, and histological features, including cellular morphology and tissue architecture, were meticulously recorded by trained pathologists to ensure accuracy and reliability of the results..

### RNA isolation and reverse transcription‑quantitative PCR (RT‑qPCR)

Tissue samples were collected from the target organs of anesthetized animals in accordance with approved ethical guidelines, with organs quickly harvested post-euthanasia to minimize RNA degradation.The collected tissues were immediately frozen in liquid nitrogen and subsequently processed using an ultrasonic cell disruptor (Branson, USA) to achieve uniform tissue grinding. The ground tissue was then subjected to RNA extraction using Trizol reagent (9112K1018; Takara Co., Ltd.) according to the manufacturer’s instructions, which included phases of homogenization, phase separation with chloroform, and RNA precipitation with isopropanol. The quality and quantity of the extracted RNA were assessed using a NanoDrop spectrophotometer.. Reverse transcription (RT) to generate cDNA was performed utilizing the Hifair@ II 1st strand cDNA synthesis kit (cat. no. 11121ES60, Yeasen Co., Ltd.) adhering strictly to the protocol provided by the manufacturer. Quantitative polymerase chain reaction (qPCR) analysis was carried out using the Hieff® qPCR SYBR Green Master Mix (NoRox) kit (cat. no. 11201ES03, Yeasen), with thermal cycling conditions comprising an initial denaturation step at 94°C for 3 min, followed by 40 cycles of 94°C for 10 sec and 60°C for 30 sec. Mean values from three independent experiments were analyzed statistically using the 2-ΔΔCq cycle threshold method. Glyceraldehyde 3-phosphate dehydrogenase (GAPDH) was utilized as an internal control, with primer sequences detailed in Table 3.

**Table 3:** Primer sequences.

Gene	Primer sequence (F)	Primer sequence (R)
SLC7A5	TACGCCTACATGCTGGAGG	GAGGGCCGAATGATGAGCAG
SLC7A11	AGGGCATACTCCAGAACACG	GGACCAAAGACCTCCAGAATG
PSAT1	CAGTGGAGCGCCAGAATAGAA	CCTGTGCCCCTTCAAGGAG
ACSF2	GAACTCGAAGTTGCAGAGTGT	GTGGCCCTGAATGTAGCTGA
ACSL4	CTCACCATTATATTGCTGCCTGT	TCTCTTTGCCATAGCGTTTTTCT
GAPDH	CCCAGCAAGGACACTGAGC AAG	GGTCTGGGATGGAAATTGTGAGGG

F, forward; R, reverse.

### Western blotting and antibodies

The tissue samples were processed in accordance with the method described in the qPCR section. Subsequently, the ground tissue was lysed using RIPA Lysis Buffer (Configured) supplemented with PMSF (cat. no. P0100; Beijing Solarbio Science & Technology Co., Ltd.), an inhibitor of serine proteases and acetylcholinesterase, while maintaining the samples on ice. The protein concentration was determined using the BCA Protein Assay kit (cat. no. C503021-0500; Biology Technology Co., Ltd.). Following this, aliquots of lysates containing 20 µg of protein were treated with sample loading buffer (cat. no. 421002; HyClone) and boiled for 5 minutes. Equal amounts of total protein (20 µg/lane) from different samples were then separated by 10% SDS‑PAGE at 120 V for 1.5 hours and transferred onto 0.22‑µm polyvinylidene difluoride membranes (cat. no. IPVH00010; Merck Millipore) at 280 mA for 1.5 hours. Subsequently, the membranes were blocked with 5% skimmed milk powder in TBS with 0.05% Tween‑20 (TBST) for 1 hour at room temperature, followed by overnight incubation with specific primary antibodies at 4˚C. The following day, the membranes were washed with TBST and incubated with HRP‑conjugated Goat Anti-Rabbit IgG [1:5,000; cat. nos. AS014; ABclonal, Inc.]. Each band was visualized using an enhanced chemiluminescence kit (ECL reagent kit; cat. no. E411-04; Nanjing Novozan Biotechnology Co., Ltd). The antibodies used, anti TUBLIN (1:3,000; cat. no. 11224–1-AP) and anti‑SLC7A5 (1:6,000; cat. no. 21865‑1‑AP), were obtained from Wuhan San Ying Co., Ltd. Finally, semi-quantitative analysis of the blots was performed using ImageJ software (version 2.14.0; National Institutes of Health).

### Immunohistochemical staining

Immunohistochemical analysis was employed to assess the expression of SLC7A5 in colon or rectal tissues. After deparaffinization with xylene and hydration with graded ethanol, paraffin sections (5 μm thick) were subjected to treatment with 3% hydrogen peroxide for 15 minutes. Subsequently, the sections were blocked with goat serum for 30 minutes at room temperature.Following the blocking step, the sections were incubated overnight at 4°C with primary antibodies targeting SLC7A5 (Sanying, Wuhan, China). On the subsequent day, the detection process continued utilizing an immunohistochemistry kit (KIT-9720, MXB). The sections were then exposed to HRP-conjugated secondary antibodies (KIT-9720, MXB) for 30 minutes at 37°C, followed by visualization using DAB chromogen solution. Hematoxylin was employed for counterstaining the nuclei. Image acquisition was carried out using the BioTek Cytation 5 (BioTek, USA), and the percentage area of positive staining was quantified using Image J software.

### Statistical analysis

Statistical analysis was performed using R software (version 4.1.2), SPSS 26.0 (IBM Corp.), and Prism 8 (Dotmatics) statistical software. For the qPCR experiment, data were analyzed using the unpaired Student’s t-test to compare expression levels between two independent groups, a method suitable for normally distributed data as verified by the Shapiro-Wilk test. The same statistical approach was applied to western blot and immunohistochemistry analyses.. Each experiment was independently repeated three times to ensure reproducibility, with a significance level set at P < 0.05. To mitigate potential biases, we randomized sample selection and employed blinding during data analysis, while also controlling for confounding variables. Sensitivity analyses were conducted to assess the robustness of our findings.

## 4 Results

### Analysis of genes associated with ferroptosis in UC

Through an exhaustive search of the GEO database, initially 5089 datasets were collected. Following stringent screening, four key chip datasets—GSE38713, GSE47908, GSE92415, and GSE87466—were selected(S1-S4 Table) offering crucial gene expression insights. Analyzing 286 samples, comprising 237 patients and 49 controls, allowed for a comprehensive understanding of ulcerative colitis mechanisms. The significance of identified hub genes was confirmed using the GSE87466 dataset, affirming their critical role in ulcerative colitis pathogenesis.

**Fig 2:**
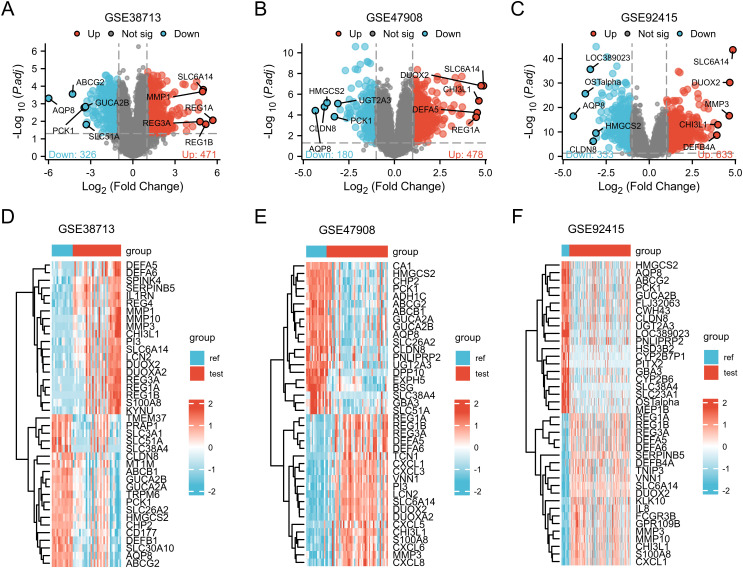
Volcano map and heat map of three UC datasets. (GSE38713, GSE47908, GSE92415) (A), (B) , (C) Volcano plot associated with dataset GSE38713, dataset GSE47908, and dataset GSE92415, showing the expression distribution of DEGs in the dataset. Based on adj. P < 0.05 and | logFC | 1 or higher standard of truncation, dot cut gene, said the red dot said raising genes. (D), (E), (F) represent the heat maps of the respective differentially expressed genes in dataset GSE38713, dataset GSE47908, and dataset GSE92415, showing the top 20 up-regulated genes and top 20 down-regulated genes in each dataset. Each column represents a sample and each row represents a gene. Red indicates upregulation and blue indicates downregulation.

**Fig 3:**
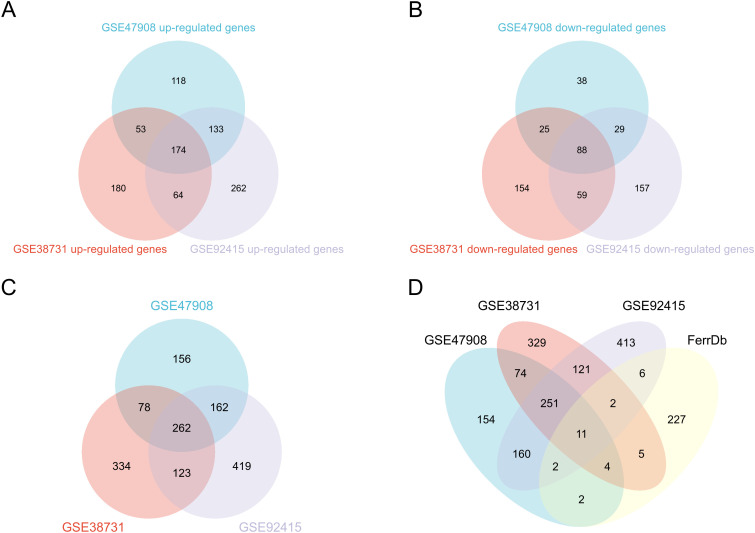
Related Venn diagram. (A) GSE38713, GSE47908, GSE92415 common up-regulated genes (B) GSE38713, GSE47908, GSE92415 common down-regulated genes (C) GSE38713, GSE47908, GSE92415 common genes (D) Venn diagram of ferroptosis-related DEGs.

Volcano plots (Fig 2A, 2B, 2C) unveiled significant changes in differential gene distribution across datasets. Heatmaps (Fig 2D, 2E, 2F) further illuminated expression trends of the top 20 upregulated and downregulated genes. Venn diagram analysis identified 262 differentially expressed genes shared among the three datasets (Table 4, S1 Table, Fig 3A, 3B, 3C), providing valuable gene expression profiles. Cross-analysis with 259 ferroptosis-related genes pinpointed 11 differentially expressed genes, visually depicted in a Venn diagram (Fig 3D), with 9 genes upregulated and 2 downregulated.

**Table 4:** The identified differentially expressed genes (DEGs).

DEGs	Gene name
Up-regulated	SLC6A14 DUOX2 CHI3L1 DEFA5 REG1A REG3A REG1B LRP8 S100A8LCN2 DEFA6 DUOXA2 MMP3 CXCL1 PI3 CXCL3 TCN1 CXCL6 MMP10 IGFBP5 FCGR3B TNIP3 CXCL13 CXCL11 CTHRC1 CXCR2 S100P CFB MMP1 IDO1 DMBT1 PRRX1 C4BPA KYNU S100A9 C4BPB IL1RN SERPINB5 KCND3 IL13RA2 CCL18 VSIG1 C2CD4A CDH3 MGP ANXA1 CD55 SERPINA3 MNDA SLC7A11 CXCL9 NOS2 LPCAT1 PCSK1 TGM2 TNC SOCS1 CSGALNACT1 PSAT1 IKBIP TRIM29 TRIB2 RGS5 CCL11 GBP1 IL33 DOK3 PECAM1 ELTD1 MMP12 PFKFB3 GUCY1B3 PHLDA1 VWF ROBO1 TMEM158 TFPI2 CD38 TIMP1 TRIM22 LAX1 GLCCI1 REG4 SLC16A14 KLHL5 PAPLN CD274 COL6A3 CDH5 SLC7A5 PF4 TMTC1 CTSK SELP ZG16B FUT8 IFITM2 TMEM45A FBXO6 SLC6A6 WNT5A GPX8 IFIT3 OSMR ECSCR CYYR1 ACSL4 COL4A1 CARD6 MYEOV CLDN1 RBPMS PDPN PIM2 F2RL2 SPP1 CHST2 GPX7 ELL2 HGF STEAP4 SDR16C5 PNOC UBE2L6 COL5A2 FCRL5 CSF2RB SPAG4 WARS LY96 SAMD9L DERL3 OLFM4 STS AGT G0 S2 SERPINA1 ANGPTL2 COL15A1 CSTA P2RY13 TFF1 FPR1 BACE2 RAB31 PLAU PITPNC1 BASP1 ISG20 BIRC3 ME1 GNA15 LAMP3 NCF2 P2RY8 SULF1 LOXL2 KLHL29 APCDD1 FSTL1 SLPI SLA HEG1 CD93 PARP8 HLA-DMA MSN ELK3 CHST15 SLCO4A1 IGFBP7 PCDH17 SERPING1 CLDN2 SRD5A3 ARFGAP3
Down-regulated	AQP8 CLDN8 HMGCS2 PCK1 UGT2A3 ABCG2 GUCA2A CHP2GUCA2B SLC26A2 ADH1C ABCB1 SLC38A4 BSG DPP10 SLC16A9 MEP1B CWH43 HSD3B2 SLC16A1 PADI2 SLC3A1 GXYLT2 TRPM6 SELENBP1 MS4A12 APOBEC3B DHRS11 SLC30A10 PAQR5 SLC19A3 RUNDC3B CNTN3 SLC4A4 SLC17A4 HEPACAM2 HSD17B2 B4GALNT2 MT1M DPP10-AS1 FMO5 PRKG2 PTGDR WDR78 ANPEP PHLPP2 LAMA1 MEP1A P2RY1 EPHX2 PPARGC1A PDE6A APPL2 PPARG GCNT2 TMED6 ISX ENTPD5 ACSF2 ANK3 MAOA TUBAL3 VLDLR TEX11 PBLD CDKN2B TSPAN7 SLC22A5 PKIB RPS6KA6 HSPB3 TMEM56 CLYBL NAAA PXMP2 ETFDH ACOX1 ADH6 CYP4F12 EDN3 ANXA13 ACVR1C RHOU VIPR1 PIGZ SLC39A5 DEFB1 MIER3 HHLA2 VAV3

**Fig 4:**
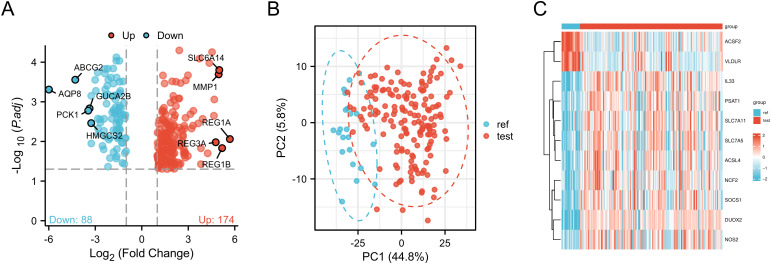
Volcano map, PC map and heat map of common differential genes and ferroptosis related genes in the three UC datasets. (A) Volcano map of DEG after removing batch effect in three datasets; Red indicates up-regulated genes and blue indicates down-regulated genes. Selection criteria for adj. P < 0.05 and | logFC | 1 or more. (B) PCA plots of degs after removal of batch effects from the three datasets. (C) Heat map of 11 differentially expressed ferroptosis-related genes between UC samples and normal samples; Red represents up-regulated genes and blue represents down-regulated genes.

A volcano plot (Figs 4A) comprehensively assessed common differentially expressed genes across the three sample groups, adhering strictly to statistical protocols. Over-PCA analysis minimized potential batch effects, ensuring data accuracy, while processed PCA plots (Fig 4B) clearly elucidated sample relationships. A heatmap (Fig 4C) vividly illustrated expression disparities of 11 ferroptosis-related genes between ulcerative colitis and normal groups, providing robust data support for further exploration of potential links between ferroptosis and ulcerative colitis.

### GO/KEGG enrichment analysis and correlation analysis were performed

**Fig 5:**
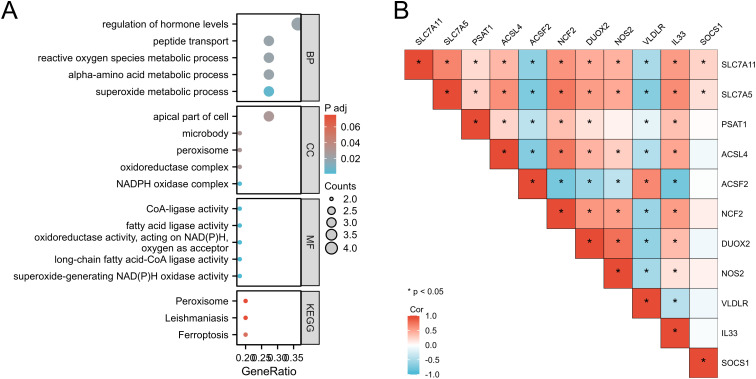
GO/ KEGG enrichment analysis and correlation analysis of 11 ferroptosis-related genes. (A) Bubble map of GO enrichment, including BP, CC and MF, and KEGG pathway enrichment. (B) Pearson correlation test of 11 ferroptosis-related genes in ulcerative colitis samples and healthy samples. *P < 0.05. BP, biological process; CC, honeycomb composition; GO, Gene ontology; KEGG, Kyoto Encyclopedia of Genes and Genomes; MF, molecular function.

For a comprehensive understanding of the biological characteristics and pathways enriched by common differentially expressed genes (DEGs), we utilized the “clusterProfiler” R package to conduct Gene Ontology (GO) and Kyoto Encyclopedia of Genes and Genomes (KEGG) analyses. The GO analysis spanned three dimensions: biological process (BP), cellular component (CC), and molecular function (MF), presenting the top five entries in each dimension visually (Table 5, Fig 5A,5B). In the BP subgroup, DEGs associated with iron toxicity demonstrated enrichments in hormone regulation, peptide transport, and oxidative stress processes. Within CC, enrichments were observed in cellular components such as microbodies and peroxisomes. MF analysis revealed activities including CoA-ligase and oxidoreductase functions. Additionally, KEGG analysis revealed enrichments in pathways such as ferroptosis and peroxisome-related processes, advancing our understanding of gene functions and regulatory mechanisms and providing a solid groundwork for further research. Pearson correlation analysis unveiled significant relationships among ferroptosis-related genes in specific disease contexts, offering insights into gene interactions.

**Table 5:** The Gene ontology/Kyoto Encyclopedia of Genes and Genomes (GO/KEGG) analysis.

Ontology	ID	Description	GeneRatio	BgRatio	pvalue	p.adjust	Ontology
BP		GO:0006801	superoxide metabolic process	3/11	72/18800	8.69e06	0.0044
BP		GO:0010817	regulation of hormone levels	4/11	496/18800	0.0001	0.0200
BP		GO:1901605	alpha-amino acid metabolic process	3/11	203/18800	0.0002	0.0200
BP		GO:0009069	serine family amino acid metabolic process	2/11	40/18800	0.0002	0.0200
BP		GO:0042554	superoxide anion generation	2/11	43/18800	0.0003	0.0200
CC		GO:0043020	NADPH oxidase complex	2/11	14/19594	2.6e05	0.0014
CC		GO:0045177	apical part of cell	3/11	424/19594	0.0015	0.0287
CC		GO:1990204	oxidoreductase complex	2/11	120/19594	0.0020	0.0287
CC		GO:0005777	peroxisome	2/11	141/19594	0.0027	0.0287
CC		GO:0042579	microbody	2/11	141/19594	0.0027	0.0287
MF		GO:0016175	superoxide-generating NAD(P)H oxidase activity	2/11	10/18410	1.46e05	0.0008
MF		GO:0004467	long-chain fatty acid-CoA ligase activity	2/11	15/18410	3.39e05	0.0008
MF		GO:0050664	oxidoreductase activity, acting on NAD(P)H, oxygen as acceptor	2/11	15/18410	3.39e05	0.0008
MF		GO:0015645	fatty acid ligase activity	2/11	22/18410	7.45e05	0.0014
MF		GO:0016405	CoA-ligase activity	2/11	27/18410	0.0001	0.0017
KEGG		hsa04216	Ferroptosis	2/10	41/8164	0.0011	0.0572
KEGG		hsa05140	Leishmaniasis	2/10	77/8164	0.0038	0.0752
KEGG		hsa04146	Peroxisome	2/10	82/8164	0.0043	0.0752

BP, Biology process; CC, Cellular component; MF, Molecular function;KEGG,Kyoto Encyclopedia of Genes and Genomes.

### PPI network generation and filtering of hub genes associated with ferroptosis

**Fig 6:**
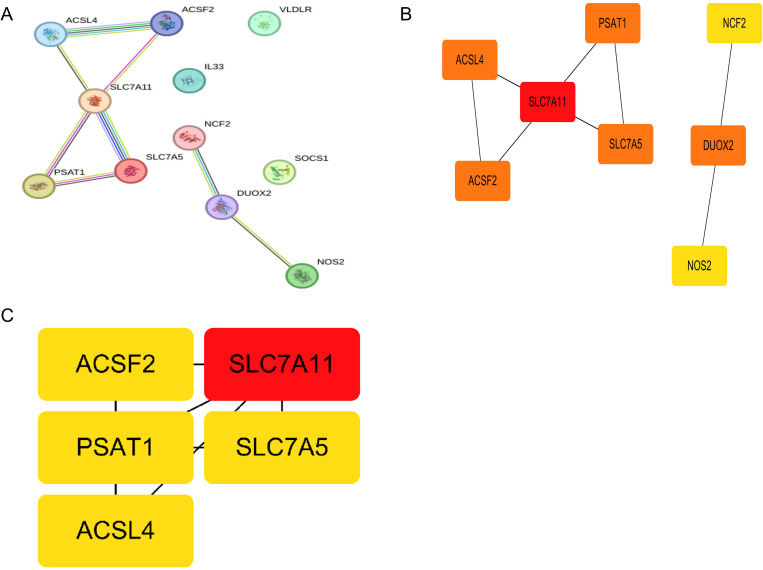
PPI analysis of the interactions between differentially expressed genes. (A) PPI network diagram of 11 iron death-related genes. (B) Eight genes used to construct the PPI network. (C) Five hub genes calculated from the PPI network.

The study utilized Protein-Protein Interaction (PPI) analysis to illustrate the relationships among the investigated genes (Fig 6A). Out of the initial 11 genes, three were excluded from the analysis due to the absence of observed interactions. Subsequently, the remaining 8 genes were used to construct the PPI network, with genes represented as nodes and their interactions depicted by connecting lines (Fig 6B). The cytoHubba plugin integrated into Cytoscape software identified five hub genes, namely SLC7A11, PSAT1, SLC7A5, ACSF2, and ACSL4 (Fig 6C), with the intensity of the node color reflecting the advanced rank order of the respective hub gene.

### Prediction results of TF and miRNA

**Fig 7:**
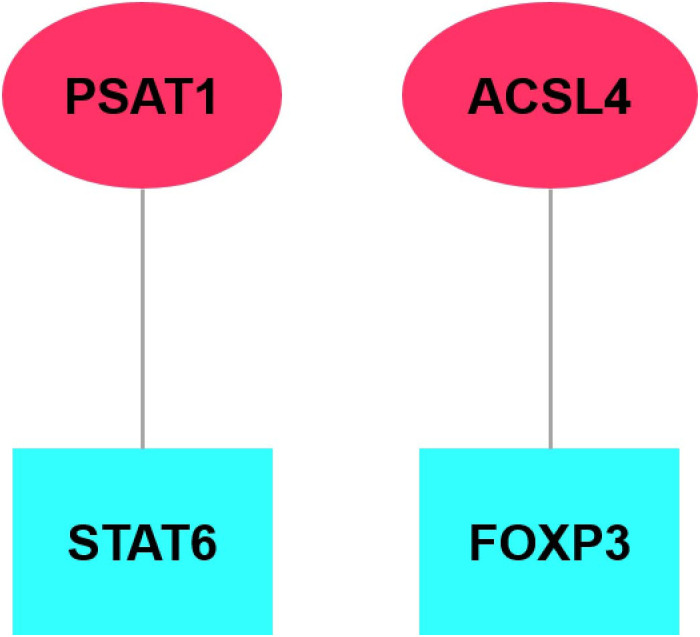
Relationship between TFs and key genes. Light blue rectangles represent TFs, and red ellipses represent Hub genes.

**Fig 8:**
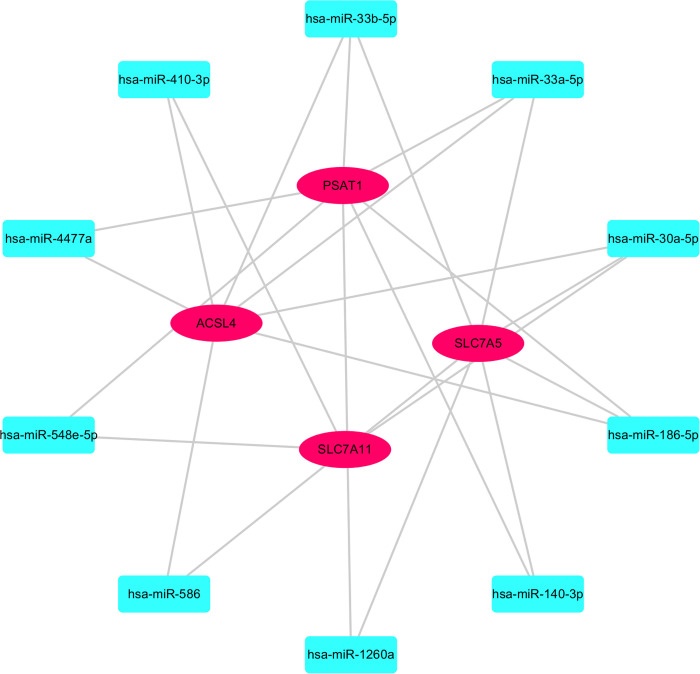
Relationship between miRNAs and key genes. Light blue rectangles represent miRNAs, and red ellipses represent Hub genes.

In the investigation of the molecular associations between ulcerative colitis (UC) and ferroptosis, a comprehensive gene regulatory network was constructed. This network integrates differentially expressed genes (DEGs), transcription factors (TFs), and microRNAs (miRNAs) associated with core genes to elucidate their interactions and regulatory functions. Analysis revealed two potential TFs—STAT6 and FOXP3—governing PSAT1 and ACSL4 (Fig 7, S6 Table), respectively. Furthermore, 506 miRNAs were identified as potential regulators of common DEGs, and an interaction network was constructed incorporating the top 10 miRNAs and DEGs (Fig 8, S7 Table), potentially impacting ferroptosis and the inflammatory processes associated with UC.

### Targeted drug prediction for related genes

The DSigDB database was employed to predict potential drugs targeting the five hub genes, with the aim of modulating ferroptosis and providing therapeutic benefits for UC. A total of 413 candidate drugs were identified(S8 Table), among which VALPROICA, pyrvinium, LY-294002, alexidine, valinomycin, astemizole, minocycline, 5707885, mepacrine, and ciclosporin emerged as the top 10 candidates. The specific rankings and corresponding statistical values are detailed in Table 6. This comprehensive drug identification strategy underscores the potential for repurposing existing therapeutics to improve clinical outcomes in patients with UC.

**Table 6:** Top 10 predicted target drugs based on five hub genes.

Drug name	P-value	Odds ratio	Combined score
VALPROICA CID CTD 0006977 pyrvinium PC3 UP LY-294002HL60 DOWN alexidine PC3 UP valinomycin PC3 UP astemizole PC3 UP minocycline HL60 UP 5707885 PC3 UP mepacrine HL60 DOWN ciclosporin PC3 UP	0.012389914 1.75E-07 1.75E-07 3.11E-07 1.03E-06 1.28E-06 4.71E-05 4.93E-05 5.61E-05 6.60E-05	58440 598.35 598.35 490.1803279 324.5054348 301.4545455 316.7142857 309.3333333 289.115942 265.9333333	256602.5919 9309.270396 9309.270396 7344.500905 4473.294297 4090.735552 3155.487949 3067.91785 2829.762918 2559.959063

### Association analysis of hub genes

**Fig 9:**
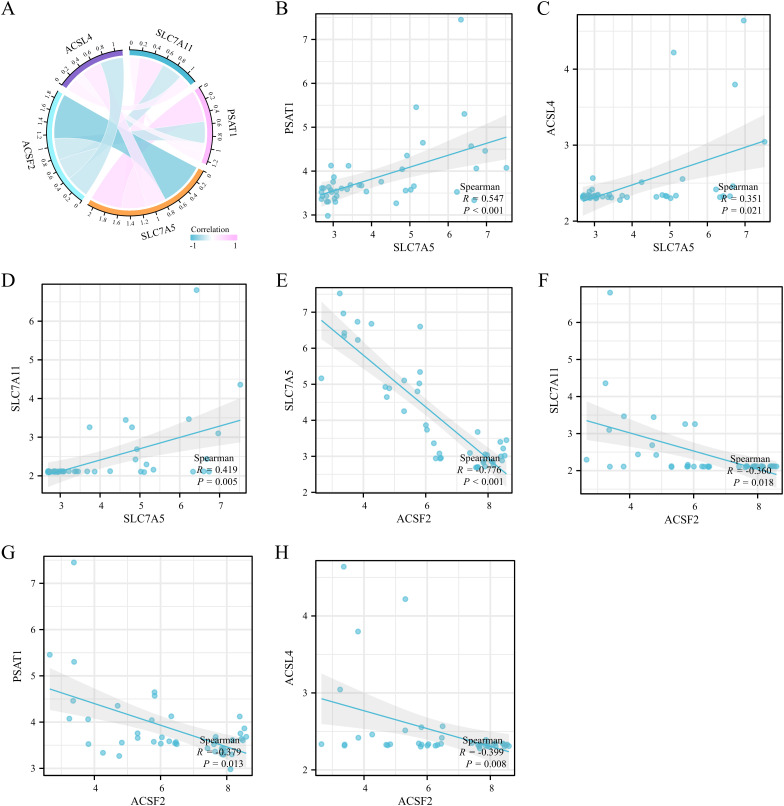
Correlations of the five hub genes. (A) Chord diagram of the correlations of the five hub genes. (B) The expression of SLC7A5 was positively correlated with PSAT1. (C) SLC7A5 was positively correlated with SLC7A11. (D) SLC7A5 expression was negatively correlated with ACSF2 expression. (E) There was a moderate negative correlation between SLC7A11 and ACSF2 expression. (F) There was a moderate negative correlation between PSAT1 and ACSF2 expression. (G) There was a moderate negative correlation between ACSL4 and ACSF2 expression.

**Fig 10:**
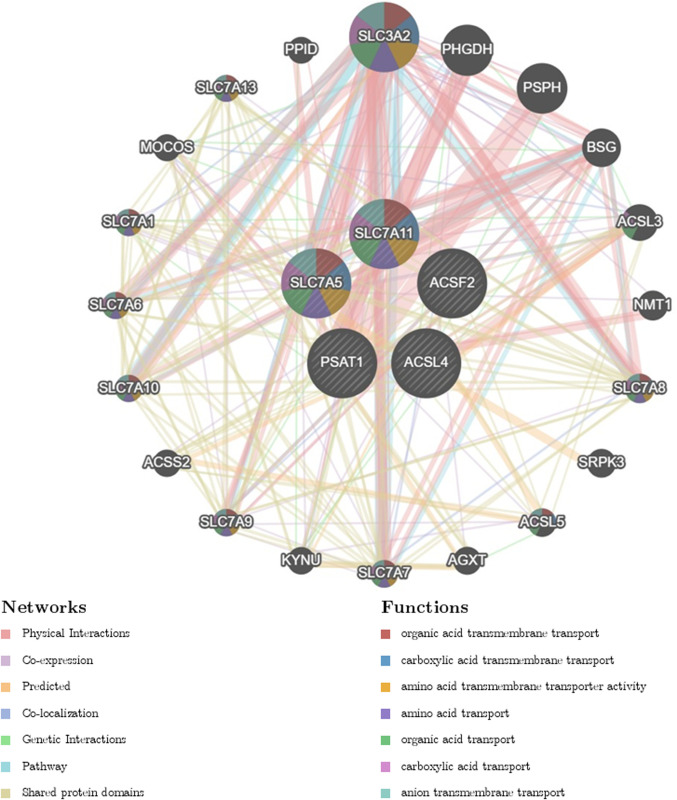
Network diagram of the five hub genes. Diagram of the five hub gene-related interaction networks identified by GeneMANIA. Network relationships and enriched functional areas are shown.

The correlation analysis among the expression levels of the five hub genes was performed (Fig 9A). Notably,SLC7A5 exhibited a significant positive correlation with PSAT1 expression (r = 0.546964663243733, p = 0.000148) (Fig 9B), as well as moderate positive correlations with ACSL4 (r = 0.351102385986107, p = 0.021) (Fig 9C) and SLC7A11 (r = 0.418922490576814, p = 0.00517) (Fig 9D). Conversely, it demonstrated a strong negative correlation with ACSF2 (r = −0.36, p = 9.74e-10) (Fig 9E), highlighting a potential regulatory relationship among these genes. Additionally, SLC7A11, PSAT1, and ACSL4 showed moderate negative correlations with ACSF2 expression (r = −0.776049531863485, p = 0.0179; r = −0.379, p = 0.0122; r = −0.399, p = 0.00797) (Fig 9F-H). GeneMANIA website analysis was employed to predict gene co-expression, functional, and physical binding characteristics of these five hub genes, which predicted co-expression, functional associations, and physical binding characteristics. The co-expressed genes were enriched in various functions, including organic acid transmembrane transport, carboxylic acid transmembrane transport, amino acid transmembrane transport activity, amino acid transport, organic acid transport, carboxylic acid transport, and anion transmembrane transport (Fig 10). These findings further support the association of hub genes SLC7A11 and SLC7A5 with inflammatory bowel disease.

### Validation of key genes was carried out through the dataset

**Fig 11:**
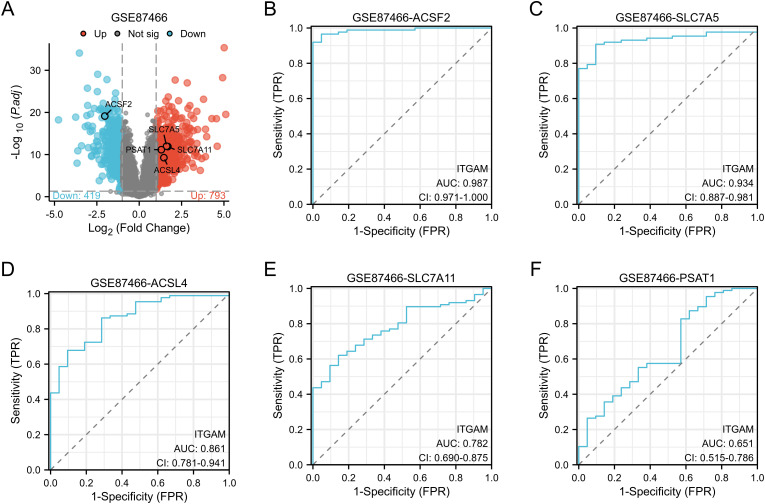
Validation of key genes in dataset GSE87466. (A) GSE87466, volcano plot shows the expression distribution of DEGs in the dataset. Based on adj. P < 0.05 and | logFC | 1 or higher standard of truncation, dot said its genes, and the red dot raised genes, SLC7A11, PSAT1, SLC7A5, ACSL4 and ACSF2 expression to display. (B-F) ROC curve analysis of central genes ACSF2, SLC7A5, ACSL4, SLC7A11 and PSAT1.

We chose the novel UC dataset GSE87466 for validation purposes. Our analysis identified a total of 751 differentially expressed genes (DEGs) in UC intestinal samples compared to control samples, comprising 419 downregulated genes and 332 upregulated genes (Fig 11A) . Consistent upregulation of SLC7A11, PSAT1, SLC7A5, and ACSL4, along with downregulation of ACSF2, was observed across datasets GSE87466, GSE38713, and GSE47908. Furthermore, receiver operating characteristic (ROC) analysis was conducted to evaluate the diagnostic efficacy of these key genes in distinguishing UC-affected tissues from normal samples. ACSF2 exhibited the highest area under the curve (AUC) value of 0.987 (Fig 11B) , indicating exceptional discriminatory accuracy for identifying UC. SLC7A5 followed closely with an AUC value of 0.934 (Fig 11C) ,suggesting its strong potential as a diagnostic marker. ACSL4, SLC7A11, and PSAT1 demonstrated promising diagnostic potential with AUC values of 0.861 (Fig 11D) , 0.782 (Fig 11E) , and 0.651 (Fig 11F) , respectively, though these values were slightly lower than those observed for ACSF2 and SLC7A5.. The results indicate that the differential expression of ACSF2 and SLC7A5 may serve as valuable biomarkers for the early diagnosis of ulcerative colitis.

### Construction and characteristics of a mouse model of DSS-induced colitis

**Fig 12:**
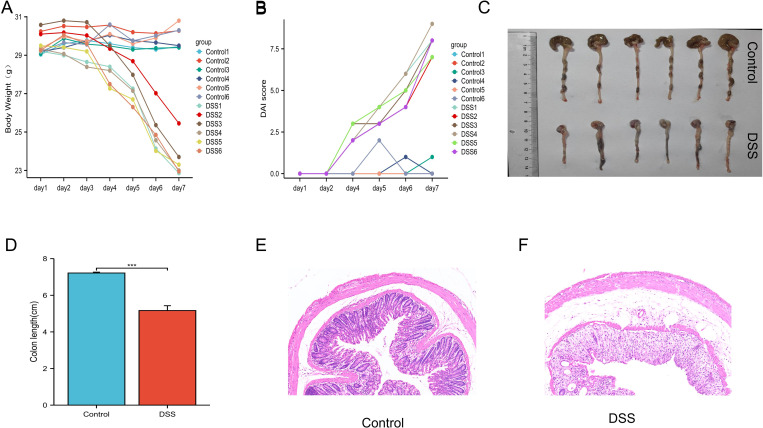
Establishment of a mouse DSS-induced UC model. (A) daily body weight changes during the experimental period; (B) DAI score of mice; (C) photos of large intestine tissues of the two groups of mice; (D) Measurements and statistics of large intestine. (E) HE staining images of normal mouse colon tissue, scale bar,100µm; (F) HE staining images of DSS mouse colon tissue, scale bar,100µm. * Represents compared to the control group. *P < 0.05, **P < 0.01, ***P < 0.001.

To investigate the selected key genes’ role in ferroptosis and their link to ulcerative colitis (UC), we induced UC in mice using 3% DSS in drinking water for 7 days while control mice received regular water. DSS-treated mice displayed typical UC symptoms, including hematochezia, altered behavior, significant weight loss, and higher DAI scores compared to controls (Fig 12A, B). Additionally, their colon length was significantly reduced (Fig 12C, D), confirming the UC model’s validity.

Colon tissue samples from both groups were analyzed using H&E staining, revealing distinct histopathological differences. UC mice exhibited evident inflammatory cell infiltration, a hallmark of UC (Fig 12E, F). These results validate our DSS-induced UC mouse model, providing a foundation for further research.

### Validation of Hub genes in UC animal samples.

**Fig 13:**
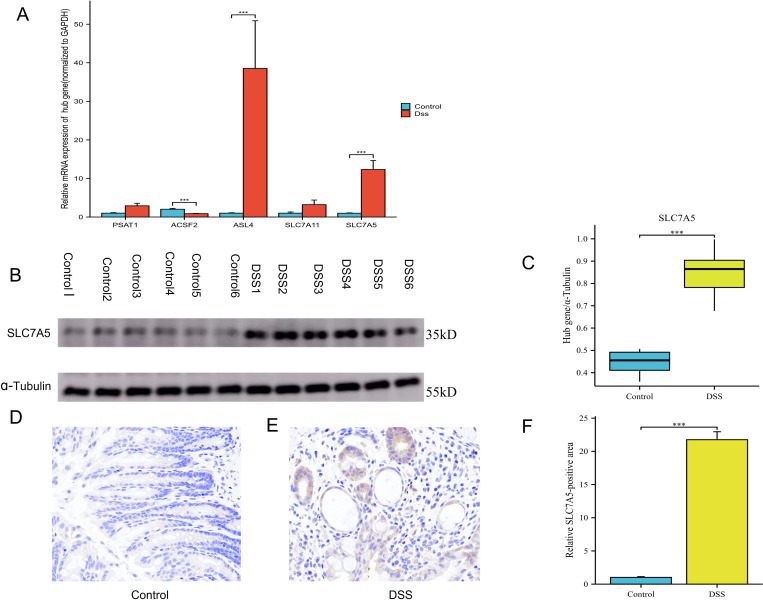
The expression of hub genes in DSS induced colitis. (A) Histogram: Comparison of RT-qPCR monitoring of HUB genes expression in the intestines of normal and UC mice; (B) Western blot analysis of SLC7A5 expression levels in the intestines of normal and UC mice; (C) Box plot: Depiction of SLC7A5 protein expression levels in the normal and DSS groups; (D) Representative image of immunohistochemical staining depicting SLC7A5 level in intestine tissue of normal mice, scale bar,20µm; (E) Representative image of immunohistochemical staining depicting SLC7A5 level in colon tissue of UC mice, scale bar,20µm; (F) Histogram: Comparison of immunohistochemical staining for SLC7A5 expression in the intestines of normal and UC mice. ***P < 0.001.

Utilizing RT-qPCR, a comparison of gene expression in colonic tissues from UC patients and healthy controls revealed significant differences in the expression of ACSF2, SLC7A5, and ACSL4, although PSAT1 and SLC7A11 exhibited consistent expression patterns, the differences were not found to be significant (Fig 13A). Subsequent Western blot analysis confirmed the elevated expression of SLC7A5, demonstrating significantly higher levels in UC tissues compared to normal tissues. Additionally, in a DSS-induced colitis model, SLC7A5 expression was also significantly increased relative to controls (P < 0.05) (Fig 13B, 13C). Immunohistochemical analysis further demonstrated increased SLC7A5 expression in DSS-induced ulcerative colitis mice, suggesting its potential role in pathogenesis (Fig 13D, 13E, 13F). These findings suggest that SLC7A5 may play a crucial role in the pathogenesis of ulcerative colitis, indicating its potential as a biomarker or therapeutic target warranting further investigation in future studies.

### Validation of the protein expression levels of SLC7A5 in UC clinical samples

**Fig 14:**
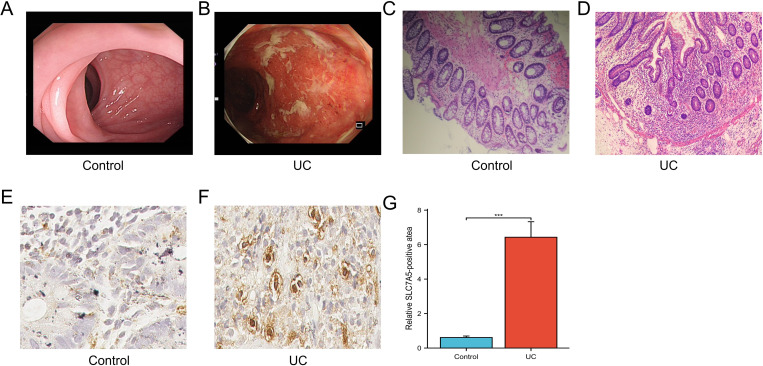
Experimental Validation of SLC7A5 in Clinical Samples. (A) Normal group intestinal histology under colonoscopy; (B) UC patient intestinal histology under colonoscopy; (C) Histological images of normal group intestinal mucosa stained with H&E, scale bar,100µm; (D) Histological images of UC patient intestinal mucosa stained with H&E, scale bar,100µm; (E) Representative image of immunohistochemical staining depicting SLC7A5 level in colon tissue of normal group, scale bar,20µm; (F) Representative image of immunohistochemical staining depicting SLC7A5 level in colon tissue of UC patient, scale bar,20µm; (G) Histogram: Comparison of immunohistochemical staining for SLC7A5 expression in the intestines of normal and UC patients. ***P < 0.001.

All clinical tissue samples from patients with UC were definitively diagnosed by two pathologists following observation under a colonoscope, tissue sectioning, and H&E staining. The clinicopathological characteristics of the patients are detailed in Table 7. The findings from colonoscopy (Fig 14A, B) and H&E staining (Fig 14C, D) are illustrated. Colonoscopy revealed mucosal ulceration with diffuse mucosal erythema and hemorrhage in the intestinal lumen, alongside multiple irregular ulcerative lesions. Microscopic examination revealed evident infiltration of inflammatory cells and lymphocytes. To validate the protein expression of the target gene SLC7A5, identified through prior animal experiments, we conducted immunohistochemistry (IHC) on both normal and UC tissues (Fig 14E, F, G). These findings were corroborated by quantitative polymerase chain reaction (qPCR) and western blot (WB) analyses performed on tissue samples from a murine model of UC, confirming the reliability and consistency of our data across different methodologies..

**Table 7:** Clinicopathological characteristics of patients with UC’s disease.

Characteristic	Age, years	Sex	Sample location
UC Patient 1	53	Female	sigmoid colon
UC Patient 2	72	Male	rectum
UC Patient 3	54	Female	sigmoid colon
UC Patient 4	64	Female	rectum
UC Patient 5	36	Male	rectum
UC Patient 6	16	Male	sigmoid colon
Control 1 Control 2 Control 3	36 41 25	Male Female Male	sigmoid colon sigmoid colon rectum
Control 4 Control 5	47 43	Female Female	sigmoid colon rectum
Control 6	56	Male	rectum

## 5 Discussion

Inflammatory bowel disease (IBD) encompasses a spectrum of chronic intestinal inflammatory disorders, exhibiting notable epidemiological variance across different regions globally [5,13,14].While historically more prevalent in North America and Europe, the incidence and prevalence of IBD in Asian populations and immigrant communities have shown a noteworthy uptrend in recent decades [15,16].Concurrently, ulcerative colitis (UC), a subtype of IBD, has emerged as a global health concern, with its impact steadily expanding [15,17,18].

The pathogenesis of UC is multifaceted, implicating aberrations in the immune system, genetic predispositions, environmental factors, apoptosis, and oxidative stress [14].Dysregulated immune responses to commensal gut microbiota precipitate sustained inflammatory cascades [19–21],culminating in mucosal damage, heightened apoptosis, oxidative stress, and exacerbated tissue injury [22].

In recent years, a growing body of research has underscored the potential involvement of ferroptosis in the pathogenesis of ulcerative colitis (UC) [23–25], This process, characterized by the accumulation of iron ions, elicits oxidative stress, mitochondrial impairment, and ultimately, cellular demise [26].Notably, iron metabolism and genes associated with ferroptosis are found to be enriched in UC patients, implicating ferroptosis as a significant player in UC pathophysiology. The accrual of iron exacerbates oxidative stress, compromises the integrity of the intestinal mucosal barrier, amplifies cellular injury and demise, thereby perpetuating a detrimental cycle of inflammation [26,27].An exhaustive exploration of the role of ferroptosis in UC is imperative for a comprehensive understanding of its pathogenesis.

In this study, we conducted a comprehensive screening of three ulcerative colitis (UC) related datasets sourced from the Gene Expression Omnibus (GEO) database. Concurrently, we employed FerrDB to compile ferroptosis-related genes, which were subsequently subjected to cross-alignment with the differentially expressed genes identified in the UC datasets. Through this rigorous analysis, we successfully identified 11 common genes of significance. These findings not only enrich our understanding of the pathogenesis of ulcerative colitis but also offer valuable insights into the development of novel therapeutic strategies targeting this debilitating condition.

Gene ontology (GO) and Kyoto Encyclopedia of Genes and Genomes (KEGG) analysis have unveiled a complex interrelation between ulcerative colitis and iron-mediated cell death. At the biological process (BP) level, there is notable enrichment observed in the regulation of hormone levels, peptide transport, and active oxygen metabolism, which offer insights into the potential implication of iron-mediated cell death in the pathogenesis of ulcerative colitis. Analysis of cellular components (CC) reveals significant enrichment in functional categories such as cell apex, microbody, and peroxisome, underscoring the importance of these cellular structures in the interplay between ulcerative colitis and iron-mediated cell death. Furthermore, at the molecular function (MF) level, there is substantial enrichment in CoA ligase activity, fatty acid ligase activity, and oxidoreductase activity, highlighting the pivotal role of these molecular functions in the association between ulcerative colitis and iron-mediated cell death. These findings hint at the potential influence of iron-mediated cell death on cellular energy metabolism, lipid metabolism, and oxidoreduction balance during the pathogenesis of ulcerative colitis.

Utilizing the KEGG database for analysis, our study has unveiled a notable enrichment of pathways related to iron-mediated cell death, peroxisome function, and leishmaniasis within the KEGG framework. This discovery offers a fresh vantage point for delving deeper into the molecular intricacies associated with ulcerative colitis and the phenomenon of iron-mediated cell death, shedding light on disease progression mechanisms. Recent investigations underscore the pivotal roles played by iron-mediated cell death and peroxisome function in disease advancement, while also highlighting the therapeutic potential of these pathways as promising targets for intervention. Thus, our findings open up new avenues for research and provide valuable insights into potential treatment modalities [9].

Subsequently, we constructed a protein interaction network utilizing differentially expressed genes. Employing the MCC algorithm, we identified five key genes—SLC7A11, PSAT1, SLC7A5, ACSF2, and ACSL4—while also predicting significant transcription factors and miRNAs. These findings furnish a pivotal reference point for future investigations and the development of therapeutic strategies.

Based on our analysis of transcription factor prediction, we elucidated the association between the genes ACSL4 and PSAT1 with the transcription factors FOXP3 and STAT6. FOXP3, a transcription factor primarily expressed in regulatory T (Treg) cells, is instrumental in orchestrating immune tolerance and regulatory mechanisms [28–31]. Its expression profile has been linked to the mitigation of autoimmune responses [32]。Considering the critical role of Treg cells in the pathogenesis of ulcerative colitis, the investigation into FOXP3 holds considerable significance [29,33]。Additionally, STAT6, identified as a pivotal regulator of ferroptosis [34,35], assumes a significant role in the context of ulcerative colitis [36,37].

Hsa-miR-186-5p is a key player in inflammatory bowel disease (IBD), ranking among the top predicted miRNAs. It regulates crucial aspects of IBD pathology, including intestinal permeability, immune response, and susceptibility to bacterial colonization by targeting genes like KRT10, FGG, and TLR4 [38]. Additionally, hsa-miR-186-5p influences fundamental cellular processes such as proliferation, migration, apoptosis, and inflammation, indicating its potential as both a tumor suppressor marker and a therapeutic target in colorectal cancer [39]. Furthermore, its involvement extends to other conditions like fragile X syndrome and cardiac transplant rejection [40,41]. The diverse regulatory roles of hsa-miR-186-5p present promising avenues for therapeutic interventions across various diseases.

In the realm of ulcerative colitis (UC) management, despite the widespread utilization of anti-inflammatory medications and immunosuppressants, high recurrence rates persist alongside notable side effects. In this study, we leveraged the DSigDB database to predict UC-associated therapeutics, unveiling minocycline as promising candidate.Shahzad et al. observed minocycline’s role in stabilizing Nrf2 by reducing ubiquitination levels, thereby impeding ferroptosis [9,42], Furthermore, minocycline demonstrates the ability to inhibit lipid peroxidation [43,44], suggesting its potential involvement in ferroptosis suppression. Minocycline offers innovative therapeutic avenues for managing UC.

In this investigation, we utilized DSS-induced UC mice to establish a reliable UC model, with results subsequently validated through RT-qPCR analysis. Notably, SLC7A5, ACSL4, and ACSF2 demonstrated significant correlations with UC pathology. Extensive research has underscored the pivotal roles of ACSF2 and ACSL4 in ferroptosis regulation. ACSL4 has been shown to actively promote ferroptosis, evidenced by notable increases in lipid peroxidation, Fe^2+^ concentration, and ACSL4 expression in inflammatory models [45,46], Conversely, down-regulation of ACSL4 expression effectively mitigates ferroptosis, offering novel therapeutic avenues for ulcerative colitis treatment [47], Moreover, investigations led by Luo and colleagues at the University of California uncovered suppressed ACSF2 expression in animal models of Salmonella typhimurium colitis and cell models. Treatment with the ferroptosis inhibitor Fer-1 successfully reversed LPS-induced alterations in ACSF2 expression in cellular contexts [48]。These findings shed light on the intricate mechanisms underlying UC pathogenesis and highlight the therapeutic potential of targeting ACSF2 in mitigating UC-associated ferroptosis.

Supported by experimental validation, this investigation reveals a heightened expression of SLC7A5 in the ulcerative colitis (UC) group compared to the normal control group, underscoring a significant association between the gene and UC pathology. This finding opens up novel avenues for the treatment of UC, presenting potential therapeutic targets. SLC7A5, also recognized as large neutral amino acid transporter 1 (LAT1), denotes a transmembrane protein facilitating the transport of large neutral amino acids, including leucine and valine, within the human system. Central to maintaining normal cellular growth and metabolism, SLC7A5 assumes pivotal roles, particularly in the proliferation and viability of tumor cells [49].

In the realm of oncology, SLC7A5 has emerged as a promising therapeutic target across various malignancies owing to its involvement in modulating amino acid equilibrium within tumor cells, thereby impacting cell proliferation and metastasis. Recent research indicates that SLC7A5 may influence ferroptosis pathways, potentially influencing the pathogenesis of psoriasis [50]. CAP-D3 deficiency may upregulate SLC7A5-SLC3a2 expression, leading to SLC7A5 protein accumulation on vesicle surfaces, enhancing mTORC1 activation, and dampening bacterial autophagy. This regulatory mechanism likely plays a pivotal role in the pathogenesis of inflammatory bowel diseases [51–54]。Furthermore, scholars including Andrew T. Schuster, Craig R. Homer, and colleagues have postulated that within intestinal epithelial cells, the SLC7A5 gene participates in amino acid transport regulation, consequently affecting mTOR localization and activation at the lysosomal membrane. CAP-D3 deficiency has been associated with heightened expression of the SLC7A5-SLC3a2 gene, leading to SLC7A5 protein accumulation on bacterial vesicle surfaces, thereby enhancing mTORC1 activation while diminishing bacterial autophagy. This regulatory mechanism likely assumes a pivotal role in the pathophysiological progression of inflammatory intestinal diseases [55].

In conclusion, the ferroptosis-associated marker SLC7A5 emerges as a pivotal focus in elucidating the mechanism underlying ulcerative colitis (UC). Investigating SLC7A5 holds promise for unraveling its role in UC pathogenesis, thereby furnishing a more precise foundation for devising personalized treatment modalities. SLC7A5 likely exerts significant influence in UC by modulating amino acid flux across intestinal cells, thereby impacting mTOR activity and intestinal barrier integrity. Furthermore, SLC7A5 may modulate immune responses in UC by regulating immune cell activity and function. Additionally, the SLC7A5 gene represents a potential target for ferroptosis-related therapeutics, offering novel avenues for drug development. Future investigations into SLC7A5’s involvement in diverse diseases, including inflammatory bowel disease, are poised to yield fresh insights and possibilities for advancing ferroptosis research applications. These findings hold potential for informing the development of personalized treatment strategies, fostering ongoing innovation, and driving progress within the medical field as a whole.

This study provides initial insights into potential transcription factors involved in the pathogenesis of ulcerative colitis (UC), specifically highlighting FOXP3, STAT6, and hsa-miR-186-5p. Our findings align with existing literature that underscores the role of immune modulation in UC; however, they also present contrasts with studies that emphasize different molecular pathways, reflecting the complexity of UC pathology. Importantly, we identified minocycline as a promising candidate for UC treatment, consistent with previous reports suggesting its anti-inflammatory properties. Despite these contributions, several limitations warrant consideration, including the preliminary nature of our findings, which necessitate further validation through larger, more rigorous studies. Additionally, the exact role of SLC7A5 in ferroptosis and its implications for UC pathophysiology remain inadequately understood, indicating a critical area for future research. To advance the understanding and treatment of UC, a multifaceted approach is essential; employing multi-omics analyses, advanced genetic technologies, and robust animal models will be crucial for elucidating the mechanisms at play. Furthermore, this study lays the groundwork for developing targeted therapeutic strategies that promise to enhance efficacy and safety in UC management. Clearly articulating these contributions bolsters the relevance of our findings, ensuring that their significance for both theoretical frameworks and clinical practice is well understood by the scientific community.

## Conclusion

This study harnessed the power of bioinformatics analysis, experimental validation, and gene expression studies to delve into the intricate relationship between ulcerative colitis (UC) and ferroptosis, yielding groundbreaking findings. Leveraging GEO database screening and GOKEGG enrichment analysis, we unraveled the complex interplay between UC and ferroptosis, shedding light on crucial biological processes and molecular functions. Moreover, the roles of transcription factors and miRNAs, notably hsa-miR-186-5p, FOXP3, and STAT6, were probed, leading to the identification of potential therapeutic drug such as minocycline. Through experimental validation in a UC mouse model, the pivotal role of the SLC7A5 gene in UC pathogenesis was underscored, offering novel insights into the disease mechanism. Future research endeavors will pivot towards further unraveling the role of SLC7A5 and other key factors, with the ultimate goal of providing precise treatments for UC patients. In summary, this study significantly advances our understanding of UC pathogenesis and presents promising avenues for future research and clinical practice.

## Supporting information

S1 Table Differentially expressed genes between healthy donors and UC patients in GSE 38713. (CSV)

S2 Table Differentially expressed genes between healthy donors and UC patients in GSE 47908. (CSV)

S3 Table Differentially expressed genes between healthy donors and UC patients in GSE 92415. (CSV)

S4 Table Differentially expressed genes between healthy donors and UC patients in GSE 87466. (CSV)

S5 Table Common DEGs in GSE38713, GSE47908, GSE92415. (XLS)

S6 Table Predicted TF-targeted DEGs interaction networks. (XLS)

S7 Table Predicted miRNA-targeted DEGs interaction networks. (XLS)

S8 Table Predicted drug-targeted DEGs interaction networks. (XLS)

## References

[1] Rogler G, et al. Extraintestinal manifestations of inflammatory bowel disease: current concepts, treatment, and implications for disease management. Gastroenterology. 2021;161(4):1118–32. 34358489 10.1053/j.gastro.2021.07.042 PMC8564770

[2] Agrawal M, Jess T. Implications of the changing epidemiology of inflammatory bowel disease in a changing world. United European Gastroenterol J. 2022;10(10):1113–20. doi: 10.1002/ueg2.12317 36251359 PMC9752308

[3] Voelker R. What is ulcerative colitis? JAMA. 2024;331(8):716. 38306113 10.1001/jama.2023.23814

[4] Ramos GP, Papadakis KA. Mechanisms of Disease: Inflammatory Bowel Diseases. Mayo Clinic Proceedings. 2019;94(1):155–65. 30611442 10.1016/j.mayocp.2018.09.013 PMC6386158

[5] Kobayashi T, Siegmund B, Le Berre C, Wei SC, Ferrante M, Shen B, et al. Ulcerative colitis. Nat Rev Dis Primers. 2020;6(1):74. doi: 10.1038/s41572-020-0205-x 32913180

[6] Bisgaard TH, Allin KH, Keefer L, Ananthakrishnan AN, Jess T. Depression and anxiety in inflammatory bowel disease: epidemiology, mechanisms and treatment. Nat Rev Gastroenterol Hepatol. 2022;19(11):717–26. doi: 10.1038/s41575-022-00634-6 35732730

[7] Liu L, Ye Y, Lin R, Liu T, Wang S, Feng Z, et al. Ferroptosis: a promising candidate for exosome-mediated regulation in different diseases. Cell Commun Signal. 2024;22(1):6. doi: 10.1186/s12964-023-01369-w 38166927 PMC11057189

[8] Wu K, et al. Ascorbic acid induces ferroptosis via STAT3/GPX4 signaling in oropharyngeal cancer. Free Radic Res. 2024;:1–13. 38385781 10.1080/10715762.2024.2320396

[9] Jiang X, Stockwell BR, Conrad M. Ferroptosis: mechanisms, biology and role in disease. Nat Rev Mol Cell Biol. 2021;22(4):266–82. doi: 10.1038/s41580-020-00324-8 33495651 PMC8142022

[10] Gao M, et al. Role of mitochondria in ferroptosis. Mol Cell. 2019;73(2):354-363.e3. 10.1016/j.molcel.2018.10.042 PMC6338496 30581146

[11] Yokote A, Imazu N, Umeno J, Kawasaki K, Fujioka S, Fuyuno Y, et al. Ferroptosis in the colon epithelial cells as a therapeutic target for ulcerative colitis. J Gastroenterol. 2023;58(9):868–82. doi: 10.1007/s00535-023-02016-4 37410250

[12] Conrad M, Lorenz SM, Proneth B. Targeting ferroptosis: new hope for as-yet-incurable diseases. Trends Mol Med. 2021;27(2):113–22. 32958404 10.1016/j.molmed.2020.08.010

[13] Segal JP, LeBlanc JF, Hart AL. Ulcerative colitis: an update. Clin Med. 2021;21(2):135–9. 10.7861/clinmed.2021-0080 PMC8002778 33762374

[14] Du L, Ha C. Epidemiology and pathogenesis of ulcerative colitis. Gastroenterol Clin North Am. 2020;49(4):643–54. 33121686 10.1016/j.gtc.2020.07.005

[15] Kuenzig ME, et al. Twenty-first century trends in the global epidemiology of pediatric-onset inflammatory bowel disease: systematic review. Gastroenterology. 2022;162(4):1147-1159.e4. 10.1053/j.gastro.2021.12.282 34995526

[16] Borowitz SM. The epidemiology of inflammatory bowel disease: Clues to pathogenesis? Front Pediatr. 2023;10:1103713. doi: 10.3389/fped.2022.1103713 36733765 PMC9886670

[17] Ng SC, Shi HY, Hamidi N, Underwood FE, Tang W, Benchimol EI, et al. Worldwide incidence and prevalence of inflammatory bowel disease in the 21st century: a systematic review of population-based studies. Lancet. 2017;390(10114):2769–78. doi: 10.1016/S0140-6736(17)32448-0 29050646

[18] Buie MJ, Quan J, Windsor JW, Coward S, Hansen TM, King JA, et al. Global Hospitalization Trends for Crohn’s Disease and Ulcerative Colitis in the 21st Century: A Systematic Review With Temporal Analyses. Clin Gastroenterol Hepatol. 2023;21(9):2211–21. doi: 10.1016/j.cgh.2022.06.030 35863682

[19] Guo XY, Liu XJ, Hao JY. Gut microbiota in ulcerative colitis: insights on pathogenesis and treatment. J Dig Dis. 2020;21(3):147–59. doi: 10.1111/1751-2980.12849 32040250

[20] Mishra S, Jha DK, Singh AK, Kumar-M P, Patil A, Sharma V. Antibiotics for induction and maintenance of remission in ulcerative colitis: systematic review and meta-analysis. Expert Rev Gastroenterol Hepatol. 2021;15(10):1215–23. doi: 10.1080/17474124.2021.1914586 33827360

[21] Zhao D, Cai C, Chen Q, Jin S, Yang B, Li N. High-Fat Diet Promotes DSS-Induced Ulcerative Colitis by Downregulated FXR Expression through the TGFB Pathway. Biomed Res Int. 2020;2020:3516128. doi: 10.1155/2020/3516128 33029504 PMC7537687

[22] Krugliak Cleveland, N., J. Torres, and D.T. Rubin, *What Does Disease Progression Look Like in Ulcerative Colitis, and How Might It Be Prevented?* Gastroenterology, 2022. 162(5): p. 1396–408. 10.1053/j.gastro.2022.01.023 35101421

[23] Huang J, Zhang J, Ma J, Ma J, Liu J, Wang F, et al. Inhibiting Ferroptosis: A Novel Approach for Ulcerative Colitis Therapeutics. Oxid Med Cell Longev. 2022;2022:9678625. doi: 10.1155/2022/9678625 35378823 PMC8976662

[24] Wu Y-T, Zhong L-S, Huang C, Guo Y-Y, Jin F-J, Hu Y-Z, et al. β-Caryophyllene Acts as a Ferroptosis Inhibitor to Ameliorate Experimental Colitis. Int J Mol Sci. 2022;23(24):16055. doi: 10.3390/ijms232416055 36555694 PMC9784863

[25] Tang D, Kroemer G, Kang R. Ferroptosis in immunostimulation and immunosuppression. Immunol Rev. 2024;321(1):199–210. doi: 10.1111/imr.13235 37424139

[26] Tang D, Chen X, Kang R, Kroemer G. Ferroptosis: molecular mechanisms and health implications. Cell Res. 2021;31(2):107–25. doi: 10.1038/s41422-020-00441-1 33268902 PMC8026611

[27] Sun Y, Chen P, Zhai B, Zhang M, Xiang Y, Fang J, et al. The emerging role of ferroptosis in inflammation. Biomed Pharmacother. 2020;127:110108. doi: 10.1016/j.biopha.2020.110108 32234642

[28] Wing JB, Tanaka A, Sakaguchi S. Human FOXP3( ) regulatory T cell heterogeneity and function in autoimmunity and cancer. Immunity. 2019;50(2):302–16. 30784578 10.1016/j.immuni.2019.01.020

[29] Wang J, Gong R, Zhao C, Lei K, Sun X, Ren H. Human FOXP3 and tumour microenvironment. Immunology. 2023;168(2):248–55. doi: 10.1111/imm.13520 35689826

[30] Ma B, Miao W, Xiao J, Chen X, Xu J, Li Y. The Role of FOXP3 on Tumor Metastasis and Its Interaction with Traditional Chinese Medicine. Molecules. 2022;27(19):6706. doi: 10.3390/molecules27196706 36235242 PMC9570879

[31] Andersen MH. FOXP3-specific immunity. Oncoimmunology. 2013;2(10):e26247. 10.4161/onci.26247 PMC3896466 24498544

[32] Katoh H, Zheng P, Liu Y. FOXP3: genetic and epigenetic implications for autoimmunity. J Autoimmun. 2013;41:72–8. doi: 10.1016/j.jaut.2012.12.004 23313429 PMC3622774

[33] Simonetti M, Yilmazer A, Kretschmer K. Genetic Tools for Analyzing Foxp3+ Treg Cells: Fluorochrome-Based Transcriptional Reporters and Genetic Fate-Mapping. Methods Mol Biol. 2023;2559:95–114. doi: 10.1007/978-1-0716-2647-4_8 36180629

[34] Yang Y, Ma Y, Li Q, Ling Y, Zhou Y, Chu K, et al. STAT6 inhibits ferroptosis and alleviates acute lung injury via regulating P53/SLC7A11 pathway. Cell Death Dis. 2022;13(6):530. doi: 10.1038/s41419-022-04971-x 35668064 PMC9169029

[35] Sheng Z, Yu Z, Wang M, Zhou R, Chen S, Yu X, et al. Targeting STAT6 to mitigate sepsis-induced muscle atrophy and weakness: Modulation of mitochondrial dysfunction, ferroptosis, and CHI3L1-Mediated satellite cell loss. Biochem Biophys Rep. 2023;37:101608. doi: 10.1016/j.bbrep.2023.101608 38188367 PMC10770525

[36] Xiong K, Deng J, Yue T, Hu W, Zeng X, Yang T, et al. Berberine promotes M2 macrophage polarisation through the IL-4-STAT6 signalling pathway in ulcerative colitis treatment. Heliyon. 2023;9(3):e14176. doi: 10.1016/j.heliyon.2023.e14176 36923882 PMC10009548

[37] Tao Y, et al. Chitosan-coated artesunate protects against ulcerative colitis via STAT6-mediated macrophage M2 polarization and intestinal barrier protection. Int J Biol Macromol. 2024;254(1):127680. 37890744 10.1016/j.ijbiomac.2023.127680

[38] Elahimanesh M, Najafi M. Cross talk between bacterial and human gene networks enriched using ncRNAs in IBD disease. Sci Rep. 2023;13(1):7704. doi: 10.1038/s41598-023-34780-x 37169818 PMC10175251

[39] Bayat Z, et al. Hsa-miR-186-5p regulates TGFβ signaling pathway through expression suppression of SMAD6 and SMAD7 genes in colorectal cancer. Biol Chem. 2021;402(4):469–80. 33938174 10.1515/hsz-2019-0407

[40] Samadishadlou M, Rahbarghazi R, Piryaei Z, Esmaeili M, Avcı ÇB, Bani F, et al. Unlocking the potential of microRNAs: machine learning identifies key biomarkers for myocardial infarction diagnosis. Cardiovasc Diabetol. 2023;22(1):247. doi: 10.1186/s12933-023-01957-7 37697288 PMC10496209

[41] Sotoudeh Anvari M, Vasei H, Najmabadi H, Badv RS, Golipour A, Mohammadi-Yeganeh S, et al. Identification of microRNAs associated with human fragile X syndrome using next-generation sequencing. Sci Rep. 2022;12(1):5011. doi: 10.1038/s41598-022-08916-4 35322102 PMC8943156

[42] Shahzad K, Bock F, Al-Dabet MM, Gadi I, Nazir S, Wang H, et al. Stabilization of endogenous Nrf2 by minocycline protects against Nlrp3-inflammasome induced diabetic nephropathy. Sci Rep. 2016;6:34228. doi: 10.1038/srep34228 27721446 PMC5056367

[43] Radad K, Amir YE, Al-Emam A, Al-Shraim M, Bin-Jaliah I, Krewenka C, et al. Minocycline protects against acrylamide-induced neurotoxicity and testicular damage in Sprague-Dawley rats. J Toxicol Pathol. 2020;33(2):87–95. doi: 10.1293/tox.2019-0066 32425341 PMC7218239

[44] Xu J, Ji J, Wang Z, Xu T. Effects of minocycline on motor function recovery and expression of glial fibrillary acidic protein and brain-derived neurotrophic factor after spinal cord injury in rats. J Pharm Pharmacol. 2021;73(3):332–7. doi: 10.1093/jpp/rgaa041 33793886

[45] Gao S, Sun C, Kong J. Vitamin D Attenuates Ulcerative Colitis by Inhibiting ACSL4-Mediated Ferroptosis. Nutrients. 2023;15(22):4845. 38004239 10.3390/nu15224845 PMC10675831

[46] Deng L, He S, Li Y, Ding R, Li X, Guo N, et al. Identification of Lipocalin 2 as a Potential Ferroptosis-related Gene in Ulcerative Colitis. Inflamm Bowel Dis. 2023;29(9):1446–57. doi: 10.1093/ibd/izad050 37000707

[47] Wang X, Quan J, Xiu C, Wang J, Zhang J. Gegen Qinlian decoction (GQD) inhibits ulcerative colitis by modulating ferroptosis-dependent pathway in mice and organoids. Chin Med. 2023;18(1):110. doi: 10.1186/s13020-023-00819-4 37649073 PMC10466729

[48] Luo L, Zhang S, Guo N, Li H, He S. ACSF2-mediated ferroptosis is involved in ulcerative colitis. Life Sci. 2023;313:121272. doi: 10.1016/j.lfs.2022.121272 36509196

[49] Kanai Y. Amino acid transporter LAT1 (SLC7A5) as a molecular target for cancer diagnosis and therapeutics. Pharmacol Ther. 2022;230:107964. 34390745 10.1016/j.pharmthera.2021.107964

[50] Mao J, Ma X. Bioinformatics Identification of Ferroptosis-Associated Biomarkers and Therapeutic Compounds in Psoriasis. J Oncol. 2022;2022:3818216. doi: 10.1155/2022/3818216 36276287 PMC9581596

[51] et al. Analysis of a new therapeutic target and construction of a prognostic model for breast cancer based on ferroptosis genes. Comput Biol Med. 2023;165:107370. 37643511 10.1016/j.compbiomed.2023.107370

[52] Ping S, Wang S, Zhao Y, He J, Li G, Li D, et al. Identification and validation of a ferroptosis-related gene signature for predicting survival in skin cutaneous melanoma. Cancer Med. 2022;11(18):3529–41. doi: 10.1002/cam4.4706 35373463 PMC9487883

[53] Huang H, Dai Y, Duan Y, Yuan Z, Li Y, Zhang M, et al. Effective prediction of potential ferroptosis critical genes in clinical colorectal cancer. Front Oncol. 2022;12:1033044. doi: 10.3389/fonc.2022.1033044 36324584 PMC9619366

[54] Li L, Gao Q, Wang J, Gu L, Li Z, Zhang S, et al. Induction of Ferroptosis by Ophiopogonin-B Through Regulating the Gene Signature AURKA in NSCLC. Front Oncol. 2022;12:833814. doi: 10.3389/fonc.2022.833814 35875069 PMC9299951

[55] Schuster AT, Homer CR, Kemp JR, Nickerson KP, Deutschman E, Kim Y, et al. Chromosome-associated protein D3 promotes bacterial clearance in human intestinal epithelial cells by repressing expression of amino acid transporters. Gastroenterology. 2015;148(7):1405-1416.e3. doi: 10.1053/j.gastro.2015.02.013 25701737 PMC4446190

